# *APOE4* Drives Sex‐ and Diet‐Dependent Effects on AD‐Like Pathology, Cognition, and Mitochondrial Function

**DOI:** 10.1096/fba.2026-00121

**Published:** 2026-05-06

**Authors:** Chelsea N. Johnson, Colton R. Lysaker, Xin Cao, Vivien Csikos, Frederick Boakye, Paul J. Kueck, Edziu Franczak, Paige C. Geiger, Jill K. Morris, John P. Thyfault, Heather M. Wilkins

**Affiliations:** ^1^ Department of Cell Biology and Physiology University of Kansas Medical Center Kansas City Kansas USA; ^2^ University of Kansas Alzheimer's Disease Center University of Kansas Medical Center Fairway Kansas USA; ^3^ Department of Neurology University of Kansas Medical Center Kansas City Kansas USA; ^4^ University of Kansas Diabetes Institute University of Kansas Medical Center Kansas City Kansas USA; ^5^ Department of Biochemistry and Molecular Biology University of Kansas Medical Center Kansas City Kansas USA

**Keywords:** Alzheimer's disease, apolipoprotein E, learning, memory, mitochondria, neurodegeneration

## Abstract

Apolipoprotein E4 (*APOE4*) is the strongest genetic risk factor for Alzheimer's disease (AD), yet it's unclear how this allele promotes disease. While factors like diet and sex may modify AD susceptibility in *APOE4* carriers, the interaction between these factors is poorly understood. Here, we sought to determine if *APOE4*, sex, and diet interact to influence AD related outcomes in mice. Male and female *APOE3* and *APOE4* targeted replacement (TR) mice were fed a low‐fat diet or high‐fat diet from 4 to 8 months old. Serum neurodegenerative disease biomarkers, brain amyloid beta (Aβ), APOE, and tau, learning and memory, hippocampal mitochondrial function and proteomics data were collected. Serum GFAP and NfL were unaffected by *APOE4*, while HFD was associated with greater serum NfL and GFAP. Whole brain Aβ was significantly altered by sex, diet, and genotype. There was a main effect of genotype on levels of brain APOE with levels being lower in *APOE4* mice. *APOE4* TR mice also exhibited impaired learning before diet. Proteomic analysis revealed that *APOE4* exerts diet‐ and sex‐dependent effects on mitochondrial pathways. This included downregulation of pyruvate metabolism in HFD males and oxidative phosphorylation in HFD females. Basal respiration was lower in *APOE4* versus *APOE3* TR females. We provide novel evidence that *APOE4* may drive early sex‐ and diet‐dependent reductions in pathways that support brain mitochondrial energy metabolism.

## Graphical abstract

APOE4 interacts with sex and diet to influence brain mitochondrial function.

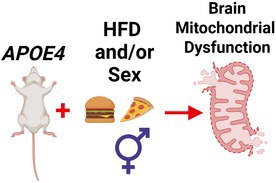

## 1 Introduction

Alzheimer's disease (AD) affects close to 7 million Americans over 65 years old and this number is projected to nearly double by 2030 [1]. Although several drugs are FDA‐approved for use in AD, including amyloid beta (Aβ)‐removing therapies that target underlying disease biology, there are no definitive methods to prevent AD development or progression. While factors such as dose and timing may explain the minimal impact of current therapeutic agents on disease course, it is also possible that pathogenic mechanisms acting upstream or independent of Aβ minimize the benefit of these drugs. Understanding alternative mechanisms that could drive disease pathology independently or in concert with Aβ may guide the identification and/or development of treatments that modify AD risk and prognosis.

Toward this goal of unraveling AD etiology and implementing effective interventions to mitigate disease risk and progression, we need to determine how known risk factors affect AD susceptibility. Apolipoprotein E4 (*APOE4*) is one of three variants of the *APOE* gene in humans and is the strongest genetic risk factor for AD [1, 2]. Conversely, *APOE2* reduces AD risk, while *APOE3* does not influence risk [1, 2]. In the brain, APOE protein is predominantly synthesized by astrocytes, but is also produced by other glial cells and neurons, particularly under stressed conditions [3]. Functionally, APOE plays an essential role in lipid transport between neurons and glial cells, which supports neuronal health, but APOE also protects against oxidative stress and may promote Aβ clearance [2, 4]. While it's unclear how APOE4 elevates AD risk, APOE4 has been linked to both loss‐of‐function protective effects as well as gain‐of‐function toxic effects relative to other APOE isoforms [2, 5, 6, 7, 8]. APOE4 is less effective at transporting lipids, protecting against oxidative stress, and removing Aβ [5, 6, 7, 8], the latter which may contribute to earlier and greater Aβ pathology in *APOE4* carriers [9, 10]. Notably, *APOE4* is also tied to reduced mitochondrial function, which has been proposed as an etiological factor in AD [11, 12]. In the absence of overt Aβ pathology, mitochondrial respiration is reduced in hippocampal and cortical tissue of *APOE4* versus *APOE3* mice [13]. Studies using neuronal cell models have further demonstrated that proteolytic cleavage products of APOE4 localize to mitochondria and directly impair respiratory function [14, 15, 16].

Since the presence of *APOE4* does not guarantee an AD diagnosis, it's important to consider how other factors may interact with this allele to promote disease. Diet may play an important and modifiable role in modulating *APOE4*‐mediated pathogenic effects. *APOE4* is the ancestral *APOE* allele in humans and in addition to AD, has been linked to a greater propensity to develop diet‐induced atherosclerosis in mouse models [17], which is more relevant in modern environments associated with Western‐style diets. Hypercholesteremia is recognized as one of 14 potentially modifiable AD risk factors, along with other factors partially tied to Western dietary habits, including obesity and diabetes [18]. While work in preclinical models provides supporting evidence that a Western diet and *APOE4* act together to exacerbate metabolic and cognitive dysfunction [19, 20, 21], less is known about changes in AD biomarkers and proteins that may reflect or influence these effects. Additionally, little is known about the impact of sex on *APOE4*‐ and diet‐mediated AD outcomes. While one study found that male *APOE4* mice may be more prone to systemic metabolic dysfunction on a high‐fat diet (HFD), molecular changes in the brain were not examined [22]. The importance of considering the potential interactive role of sex with *APOE4* and diet is supported by evidence that AD risk is stronger in female versus male *APOE4* carriers [23] and knowledge that lipid metabolism differs considerably between sexes.

Here, we used a mouse model to determine if *APOE* genotype, Western‐style diet, and sex interact to influence AD‐related outcomes. We used *APOE3* (control) and *APOE4* targeted replacement (TR) mice fed a low‐fat diet (LFD) or high‐fat diet (HFD) with matching sucrose for 4 months and assessed AD biomarkers in blood and brain tissue as well as spatial learning and memory using the Barnes maze protocol. We also evaluated the proteome and mitochondrial respiration in brain tissue. Our hypothesis was that *APOE4*, HFD, and female sex would be linked to the most pronounced derangements in AD pathology, cognitive function, and metabolic outcomes. We found that *APOE4* was associated with significant diet‐ and sex‐dependent proteomic effects in hippocampal enriched coronal tissue while affecting markers of neurodegenerative disease. Mitochondrial pathways were among the top biological processes affected by *APOE4* in all groups, and this was associated with altered mitochondrial function in females, supporting existing literature that mitochondria may play a central role in *APOE4*‐driven disease.

## 2 Methods

### 2.1 Animals

Male and female *APOE3* and *APOE4* TR mice on a C57BL/6NTac background were used in this study (*n* = 61 mice, 7–8 mice per genotype/diet/sex combination). Mice were purchased from Taconic and maintained in groups of three to four in the animal facility at the University of Kansas Medical Center (KUMC). Until 4 months old, mice were fed a standard chow diet (14% kcal fat, Teklad Global Rodent Diets, 8604). Mice were then switched to a low‐fat diet (LFD, D12110704, 10% kcal fat, Research Diets, New Brunswick, NJ) or high‐fat diet (HFD, D12451, 45% kcal fat, Research Diets) until they were sacrificed at 8 months old. Diet groups were chosen at random ensuring equal sex distribution and weight prior to diet initiation. The LFD and HFD had matching sucrose at ~17% kcal. At 6 months of age (2 months post diet), blood was collected via tail vein, placed into an Eppendorf tube, and centrifuged for serum collection. Phenobarbital (0.5 mg phenobarbital/g body weight) was used to anesthetize mice, which was followed by tissue collection. Animal procedures used in this study were approved by the Institutional Animal Care and Use Committee at KUMC.

### 2.2 Tissue Collection

Cardiac blood was drawn into a syringe and placed into an Eppendorf tube. Blood was centrifuged (4°C, 10 min, 7000 *g*) and the supernatant containing the serum was transferred to a new tube and stored at −80°C for serum analyses. During blood processing, the brain was removed. The left hemisphere of the brain was weighed and placed in a 50 mL conical tube containing 8 mL of ice‐cold isolation buffer A (320 mM sucrose, 10 mM Tris‐Cl, 1 mM EDTA, 2.5 g/L BSA, pH 7.4) for respirometry. The right hemisphere of each mouse brain was dissected using a brain matrix. To obtain a section containing the hippocampus, including the dentate gyrus, a razor blade was placed at the approximate anterior boundary of the hippocampus (Figure S1). This single hippocampal coronal section was extracted from each brain and stored at −80°C for proteomics. The remainder of the whole brain was stored at −80°C for downstream biochemical assays.

### 2.3 Serum Biomarkers

Glial fibrillary acidic protein (GFAP) and neurofilament light chain (NfL) were measured in serum using the Neurology 2‐Plex B Advantage Kit (103,520, Quanterix Simoa HD‐X, Billerica, MA) per the manufacturer's instructions.

### 2.4 Western Blot Analysis

Levels of total tau and phosphorylated tau were determined using western blot analysis. Right hemisphere brain samples were lysed using ice‐cold homogenization buffer (1% Triton X‐100, 50 mM HEPES, 12 mM Na pyrophosphate, 100 mM NaF, 10 mM EDTA, protease inhibitor, phosphatase inhibitor) and centrifuged at 4°C for 25 min at 15,000 *g*. A BCA assay (ThermoFisher) was used to quantify protein content in the supernatant. An equal amount of protein was resolved on 4%–15% Criterion TGX gels (Bio‐Rad). Gels were subsequently transferred to PVDF membranes (Bio‐Rad) and blocked for 1 h at room temperature (RT) in 5% bovine serum albumin (BSA) in 1X phosphate buffered saline with Tween (PBST). Membranes were incubated overnight in primary antibodies (1:1000 dilution) at 4°C with gentle agitation. Blots were then washed three times in 1X PBST before being incubated in secondary antibody (1:2000 dilution in 5% non‐fat dry milk) for 1 h at RT. Membranes were washed three more times in 1X PBST before being imaged using SuperSignal West Dura ECL substrate (ThermoFisher) on a ChemiDoc XRS imaging system (Bio‐Rad) and Amido Black was used to stain for total protein. Primary antibodies used in this study included Total Tau (Abcam, ab76128), pTau181 (Abcam, ab254409), and pTau396 (Invitrogen, 44‐752G).

### 2.5 Enzyme Linked Immunosorbent Assays

Amyloid beta (Aβ) and apolipoprotein E (APOE) levels were measured using commercially available ELISA kits. Briefly, right hemisphere mouse brains were lysed as described above. Supernatants were then diluted using kit‐provided dilution buffers at recommended concentrations. Specific ELISA kits included mouse Aβ 1–40 (Invitrogen, KMB3481), mouse Aβ 1–42 (Invitrogen, KMB3441), and human APOE (Invitrogen, EHAPOE). Assays were completed as instructed in the manufacturer's protocol and final concentrations were normalized to protein content as determined by a BCA assay (ThermoFisher).

### 2.6 Barnes Maze

The Barnes maze arena consisted of twenty 5.0 cm holes evenly spaced along the outside of a 1.0 m diameter circular table. One hole was designated as the target hole and consisted of a dark box. Four visual cues were placed around the maze. For initial acclimation, a subset of mice (*n* = 3–4 per genotype/sex/diet combination) were placed inside the target box outside of the Barnes maze arena for 2 min. The following day, the same mice were placed in the middle of the Barnes maze table under a cup. After approximately 20 s, the cup was lifted to release the mouse. Each mouse was allotted 180 s to locate and move into the target box. If the mouse failed to enter the target box within 180 s, the observer guided the mouse to the goal. This was repeated four times per day for five consecutive days. Within‐day trials were repeated within 5–10 min of the previous trial. Each five‐day training session was repeated at three ages: 3, 6, and 8 months. A new Barnes maze set up, with varied placement of the target hole and visual cues, was used for each training session. Memory retention of the Barnes maze set up was assessed in the same subset of mice at 6 months old (recall of arena at 3 months old) and 8 months old (recall of arena at 6 months old).

### 2.7 Protein Isolation From Hippocampal Coronal Sections

Hippocampal coronal tissue from the right brain hemisphere of a subset of mice (*n* = 4 per genotype/sex/diet combination) was crushed with a pestle and placed in a liquid‐nitrogen cooled tube. Ice‐cold homogenization buffer (1% Triton X‐100, 50 mM HEPES, 12 mM Na pyrophosphate, 100 mM NaF, 10 mM EDTA, protease inhibitor, phosphatase inhibitor) was immediately added to the tissue. A stainless‐steel bead was then added, and samples were beat on a TissueLyser II (Qiagen, Germantown, MD) programmed to beat samples at 20 Hz for 2 min. Samples were beat three times and placed on ice for 10 min between each set. Homogenized samples were then centrifuged (4°C, 25 min, 15,000 *g*) and the supernatant containing the proteins was transferred to a new tube. Protein content was measured with a bicinchoninic acid assay (Thermo Fisher, Waltham, MA) and 50 μg of lysed protein was aliquoted into a new tube for proteomics at the University of Arkansas for Medical Sciences (UAMS).

### 2.8 Proteomics

Proteins isolated from hippocampal coronal sections were processed for mass spectrometry at UAMS. All samples were completed in the same batch. Proteins were reduced, alkylated, and extracted with a chloroform/methanol mixture. Porcine trypsin (Promega) was then used to digest proteins into peptides. Reverse phase XSelect CSH C18 2.5 μm resin (Waters) on an in‐line 150 × 0.075 mm column using an Ultimate 3000 RSLCnano system (Thermo) was used to separate tryptic peptides. This was followed by peptide elution using a 60 min gradient from 98:2 to 65:35 buffer A (0.1% formic acid, 0.5% acetonitrile) to buffer B (0.1% formic acid, 99.9% acetonitrile) ratio. Eluted peptides were ionized by electrospray (2.2 kV) and analyzed on an Orbitrap Exploris 480 mass spectrometer (Thermo). Following each data‐independent acquisition (DIA) duty cycle, precursor spectra were acquired spanning the m/z range of the gas‐phase fraction (i.e., 496–602 m/z, 60,000 resolution, normalized AGC target 100%, maximum injection time 50 ms). The mass spectrometer was set to acquire a precursor scan (385–1015 m/z, 60,000 resolution, normalized AGC target 100%, maximum injection time 50 ms) for wide‐window acquisition, followed by 50× 12 m/z DIA spectra (12 m/z precursor isolation windows at 15,000 resolution, normalized AGC target 100%, maximum injection time 33 ms).

### 2.9 Mitochondrial Isolation

Immediately following collection, the entire left hemisphere of the brain was weighed and placed in a conical tube filled with 8 mL of isolation buffer A (320 mM sucrose, 10 mM Tris‐Cl, 1 mM EDTA, 2.5 g/L BSA, pH 7.4). Brain tissue was then transferred to a pre‐chilled glass/Teflon potter and homogenized with 4–6 strokes at 1000 rpm. Homogenized brain tissue was transferred back to the original conical tube and centrifuged (4°C, 10 min, 1000 *g*). The supernatant containing mitochondria was then transferred to a new tube and centrifuged again (4°C, 10 min, 10,000 *g*). The supernatant was discarded and the pellet containing mitochondria was resuspended in 6 mL of isolation buffer B (320 mM sucrose, 10 mM Tris, 1 mM EDTA, pH 7.4). Resuspended mitochondria were centrifuged (4°C, 10 min, 10,000 *g*) and the supernatant was discarded. The mitochondrial pellet was resuspended in isolation buffer B. The volume of buffer B used for resuspension was adjusted based on a 1:8 ratio (initial brain weight: volume buffer B), such that 8 μL of buffer B was added per mg of tissue. The volume of buffer B used for resuspension was adjusted to a ratio of 8 μL of buffer per mg of tissue (based on initial brain weight).

### 2.10 Mitochondrial Respiration

Isolated brain mitochondria were used to assess mitochondrial oxygen flux on the Oroboros Oxygraph‐2k system (Innsbruck, Austria). Basal respiration was measured in the presence of 2 mM malate, 0.01 mM CoA, 2.5 mM carnitine, and 5 mM potassium pyruvate. State 3 respiration was determined by adding 2.5 mM ADP. Complex I‐linked respiration was then measured in the presence of previous substrates and 2 mM glutamate (state 3 + glutamate). Complex II‐linked respiration was interrogated by adding 10 mM succinate (state 3S). Finally, FCCP was added sequentially in steps (0.00015 mM each time) until maximal respiration was reached. Average steady state oxygen flux was determined for each mitochondrial respiratory state.

### 2.11 Statistical Analysis

#### 2.11.1 Alzheimer's Disease Biomarker/Pathology and Mitochondrial Respiration Data

Grubbs' test was used to identify and remove outliers using GraphPad prism 10.1.2. Three‐way ANOVA was used to assess for an interaction effect between *APOE* genotype, sex, and diet on Alzheimer's disease biomarkers and mitochondrial respiration. If the three‐way ANOVA was significant, Fisher's least significance difference (LSD) multiple comparisons were performed for all *APOE* genotype, sex, and diet combinations. If the three‐way ANOVA was not significant, two‐way ANOVA interactions were interpreted. If any two‐way ANOVA interactions were significant, corresponding multiple comparisons were evaluated with Fisher's LSD. If there were not any significant two‐way ANOVA interactions, main effects of *APOE* genotype, sex, and diet were interpreted. Significance was set at alpha 0.05. ANOVA analyses were performed in SPSS 29.0.

#### 2.11.2 Barnes Maze Data

Repeated measures analyses were used to compare latency and distance traveled to goal between groups during each training period. Three‐way ANOVA was used to determine the effects of *APOE* genotype, sex, and diet on latency and distance at recall timepoints. To account for mice that did not escape the maze in the allotted 180 s, Kaplan–Meier curves of escape percentage as a function of escape latency were compared using the log‐rank Mantel‐Cox test as previously suggested [24]. If the curves were significantly different (*p* ≤ 0.05), pairwise log‐rank comparisons were used to compare escape distributions among groups. Significance was set at alpha 0.05 and adjusted for pairwise comparisons by dividing 0.05 by the number of comparisons tested. Analyses were performed in SPSS 29.0.

#### 2.11.3 Proteomics Data

Spectronaut (Biognosys version 18.3) was used to cross match proteomics data with the UniProt *Mus musculus* database. DirectDIA was used with an identification precursor and protein q‐value cutoff of 1%, generate decoys set to true, the protein inference workflow set to maxLFQ, inference algorithm set to IDPicker number, quantity level set to MS2, cross‐run normalization set to false, and the protein grouping quantification set to median peptide and precursor quantity. ProteiNorm [25] was used to evaluate protein MS2 intensity values and data was normalized with variance stabilization normalization (VSN) [26]. ProteoDA was used for statistical analyses using Linear Models for Microarray Data (limma) with empirical Bayes (eBayes) smoothing to the standard errors [27, 28]. Proteins with *p* ≤ 0.05 for between group comparisons were considered statistically different; no false discovery rate was applied. Ingenuity Pathway Analysis (IPA, Qiagen) was used to determine pathway enrichment of all detected proteins and proteins that significantly differed in *APOE4* versus *APOE3* TR mice, with an enrichment *p* value threshold of *p* ≤ 0.05. IPA was also used to predict the direction of pathway activity using fold‐change data for significantly altered proteins, with an activation *z*‐score cut‐off of ±2. MitoCarta3.0 was used to identify proteins in our dataset that localize to mitochondria [29]. Percent mitochondrial protein enrichment was calculated by dividing the summed intensity of all mitochondrial proteins in our dataset by the summed intensity of all proteins in our dataset and multiplying by 100 for each sample. We repeated pathway analyses of significantly altered proteins in our mitochondrial‐filtered data using IPA.

## 3 Results

### 3.1 Alzheimer's Disease Biomarkers Are Affected by Apolipoprotein E4 ( *APOE4* ) and Are Increased by High‐Fat Diet (HFD) and Male Sex

The mice examined in this study were also examined in a previously published study, where effects of diet, sex, and genotype on weight, muscle, and lean mass, and body composition were presented [30].

**FIGURE 1:**
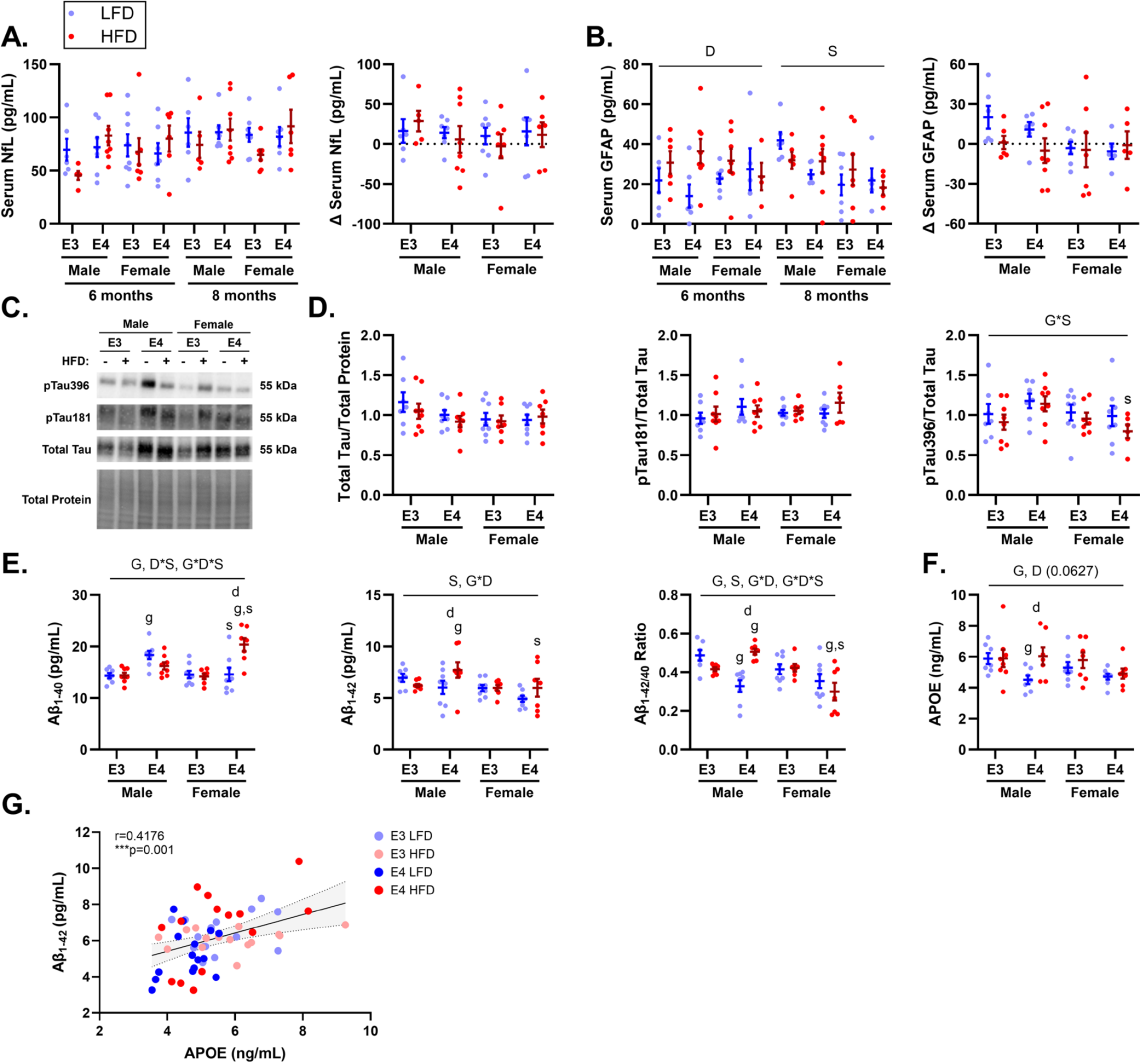
Alzheimer's disease biomarkers are affected by apolipoprotein E4 (*APOE4*) and are increased by high‐fat diet (HFD) and male sex. Neurofilament light chain (NfL) (A) and glial fibrillary acidic protein (GFAP) (B) were measured in mouse serum at 6 and 8 months of age. Change in biomarker levels were calculated by subtracting six‐month‐old serum values from eight‐month‐old serum values. Representative western blots for brain phosphorylated and total tau in 8‐month‐old mice (C). Densitometry quantification of total Tau, pTau181, and pTau396 in 8‐month‐old mice (D). Brain amyloid levels including Aβ40, Aβ42, and their ratio in 8‐month‐old mice (E). Quantification of brain APOE protein levels in 8‐month‐old mice (F). Pearson correlation analysis of Aβ42 vs. APOE in *APOE3* and *APOE4* TR mice in 8‐month‐old mice (G). Values are presented as mean ± SEM. The following symbols represent significant (*p* ≤ 0.05) comparisons: D, main effect diet; S, main effect sex. G, main effect genotype; G*S, genotype by sex interaction; D*S, diet by sex interaction; G*D, genotype by diet interaction; G*D*S, genotype by diet by sex interaction; s, sex effect within genotype/diet condition; g, genotype effect within diet/sex condition; d, diet effect within genotype/sex condition. Sample size: *n* = 5–8/group. LFD, low‐fat diet; HFD, high‐fat diet; E3, apolipoprotein E3; E4, apolipoprotein E4.

We assessed serum NfL and GFAP, markers of neurodegeneration and astrocyte reactivity respectively, at 6 and 8 months old. Serum NfL values at 6 months old, 8 months old, and change between these timepoints were unaffected by *APOE* genotype, sex, and diet (Figure 1A). In contrast, serum GFAP was significantly greater in HFD versus LFD mice at 6 months old (main effect diet, *p* = 0.047) and in male versus female mice at 8 months old (main effect sex, *p* = 0.007) (Figure 1B). However, change in GFAP was not impacted by *APOE* genotype, sex, or diet. Since obesity may impact circulating levels of NfL and GFAP due to differences in blood volume [31, 32, 33], we also evaluated these biomarkers with body weight as a co‐variate in our model (Table 1). We found that body mass was a significant co‐variate for NfL at 6 months and change in GFAP over time. After accounting for this effect, HFD was associated with greater NfL levels at 6 months old (main effect diet, *p* = 0.050) while sex and diet significantly affected change in GFAP levels. Over the course of 2 months, GFAP levels dropped in HFD mice while increasing in LFD mice (main effect diet, *p* = 0.008) and decreased in females while rising in males (main effect sex, *p* = 0.007).

**TABLE 1:** ANCOVA results for analyses of serum AD biomarkers.

	ANCOVA *p* values							Effect of co‐variate		Estimated marginal means			
	G	D	S	G × D	G × S	D × S	G × D × S	Body mass co‐variate *p* value	Body mass co‐variate partial	LFD	HFD	Male	Female
NfL, 6 months	0.111	0.050*	0.337	0.119	0.113	0.390	0.828	0.010*	0.141	58.939 [6.180]	80.531 [6.591]	N/A	N/A
NfL, 8 months	0.168	0.939	0.582	0.149	0.772	0.936	0.575	0.744	0.002	N/A	N/A	N/A	N/A
Change NfL	0.878	0.899	0.536	0.820	0.273	0.643	0.501	0.819	0.001	N/A	N/A	N/A	N/A
GFAP, 6 months	0.844	0.009*	0.362	0.809	0.775	0.209	0.268	0.082	0.076	15.881 [4.276]	35.347 [4.068]	N/A	N/A
GFAP, 8 months	0.137	0.734	0.011*	0.827	0.663	0.630	0.101	0.654	0.005	N/A	N/A	32.948 [2.922]	20.657 [3.196]
Change GFAP	0.249	0.008*	0.007*	0.384	0.689	0.878	0.789	0.014*	0.146	10.458 [4.622]	−9.170 [4.614]	9.719 [4.230]	−8.431 [4.493]

*Note:* ANCOVA *p* values for serum neurofilament light chain (NfL) and glial fibrillary acidic protein (GFAP) are shown for main effects of genotype (G), diet (D), and sex (S), and for interaction between genotype/diet (G × D), genotype/sex (G × S), diet/sex (D × S), and genotype/diet/sex (G × D × S) when controlling for body mass. Estimated marginal means [SEM] are only shown for significant comparisons.

*p < 0.05.

Abbreviations: HFD, high‐fat diet; LFD, low‐fat diet.

We also measured AD‐like pathology in the brains of these mice to identify any potential interactions between sex, diet, and *APOE* genotype. While no differences were observed in total Tau or pTau181, we found a genotype by sex effect for pTau396, with a trend toward higher levels in *APOE4* TR males compared to *APOE3* males (*p* = 0.058) (Figure 1C,D). Additionally, HFD *APOE4* TR females had significantly lower pTau396 levels than HFD *APOE4* TR males (*p* = 0.0258) (Figure 1C,D).

Next, we examined levels of Aβ peptides 1–40 and 1–42. Interestingly, *APOE4* TR mice exhibited higher levels of Aβ40 than *APOE3* TR mice (main effect genotype, *p* ≤ 0.0001) (Figure 1E). We also observed significant diet by sex (*p* = 0.0032) and diet by sex by genotype (*p* = 0.0011) interactions (Figure 1E). Specifically, male *APOE4* TR mice on a LFD showed elevated Aβ40 levels when compared to their male *APOE3* LFD (*p* = 0.0018) and female *APOE4* LFD (*p* = 0.0024) counterparts (Figure 1E). In female HFD *APOE4* TR mice, Aβ40 levels were elevated relative to their male *APOE4* HFD (*p* = 0.0013), female *APOE3* HFD (*p* < 0.0001), and female *APOE4* LFD (*p* < 0.0001) counterparts (Figure 1E). Analysis of Aβ42 revealed significantly higher levels in males than in females (main effect of sex, *p* = 0.0075) (Figure 1E). There was an additional main effect of genotype by diet (*p* = 0.0205) where HFD *APOE4* TR males had increased levels of Aβ42 when compared to *APOE4* males on a LFD (*p* = 0.0214) and *APOE3* males on a HFD (*p* = 0.0408) (Figure 1E). Calculation of the Aβ42/40 ratio revealed lower values in *APOE4* TR mice than *APOE3* TR mice (main effect genotype, *p* = 0.0032) and in females compared to males (main effect sex, *p* = 0.0041) (Figure 1E). We also observed that a HFD increased the ratio in *APOE4* TR male mice compared to *APOE4* TR LFD males (*p* ≤ 0.0001), *APOE3* TR HFD males (*p* = 0.035), and *APOE4* TR HFD females (*p* ≤ 0.0001) (Figure 1E). There were additional genotype by diet (*p* = 0.0251) and genotype by diet by sex interactions identified (*p* = 0.0004) (Figure 1E).

Given its role in amyloid clearance, APOE protein levels were quantified in the brains of these mice. We found that there was a main effect of genotype where *APOE4* TR mice had lower brain APOE than *APOE3* TR mice (*p* = 0.0255) (Figure 1F). Unsurprisingly, we also found that there was a trend for a main effect of diet where HFD animals had higher APOE protein levels than their LFD counterparts (*p* = 0.0627) (Figure 1F). When performing correlation analysis of Aβ42 and APOE, we found no relationship in *APOE3* TR mice, whereas *APOE4* TR animals exhibited a positive correlation, with higher Aβ42 levels associated with increased APOE (Figure 1G).

### 3.2 Spatial Learning Is Impaired in Three‐Month‐Old Apolipoprotein E4 ( *APOE4* ) TR Mice

**FIGURE 2:**
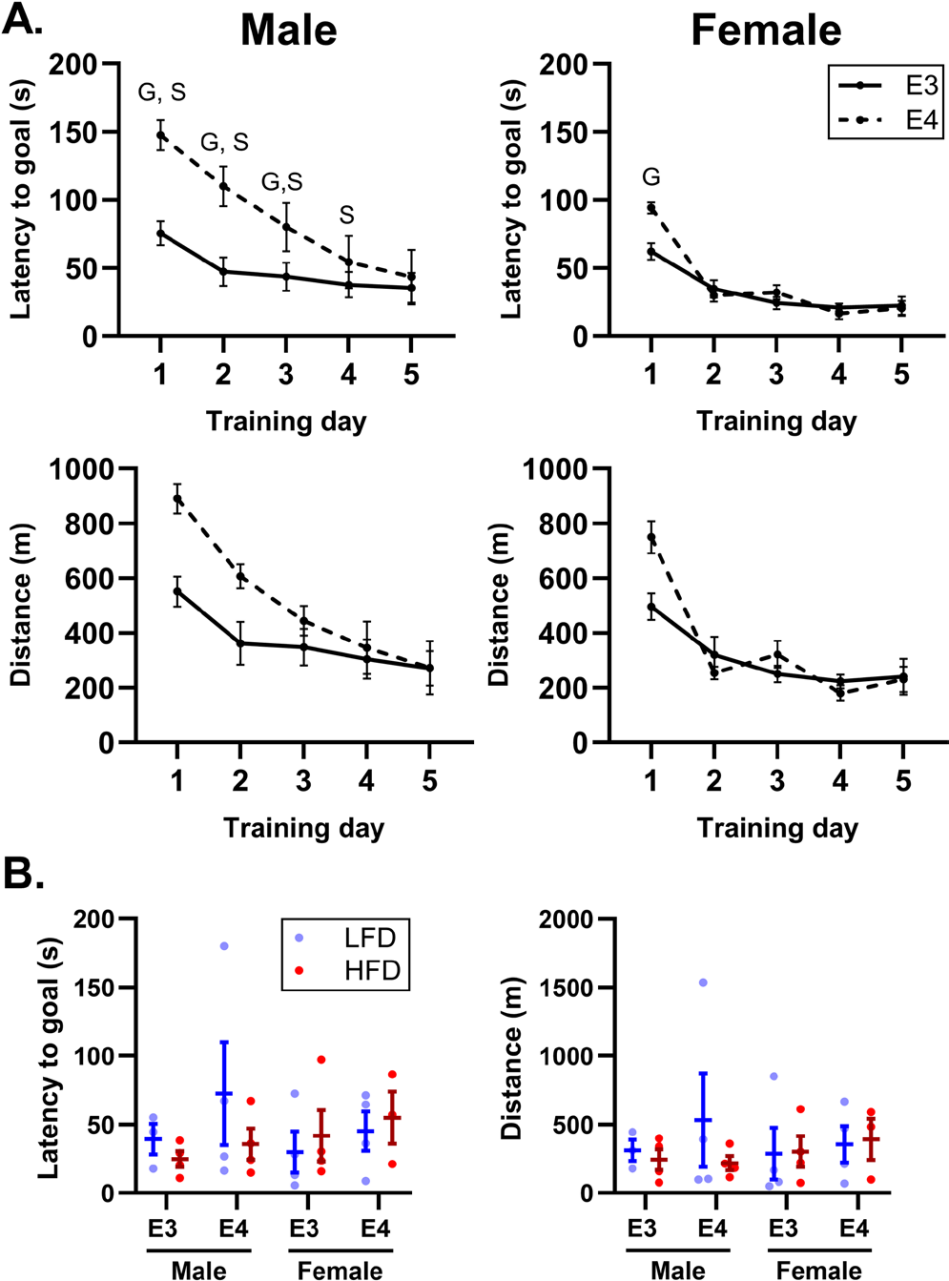
Spatial learning is impaired in three‐month‐old apolipoprotein E4 (*APOE4*)‐targeted replacement (TR) mice. Spatial learning was evaluated with the Barnes maze procedure before diet started at 3 months of age. Latency to goal and distance traveled are presented as mean ± SEM for each training day at 3 months of age. (A) Recall of the target hole to assess memory was tested at 6 months of age, 2 months after diet initiation (B). The following symbols represent significant (*p* ≤ 0.05) comparisons: G, genotype effect within sex; S, sex effect within genotype. Sample size: *n* = 3–4/group. LFD, low‐fat diet; HFD, high‐fat diet; E3, apolipoprotein E3; E4, apolipoprotein E4.

At 3 months old before diet start, repeated measures analyses showed a trending interaction between *APOE* genotype, sex, and training day on latency to goal (*p* = 0.061) during the Barnes maze procedure (Figure 2A). Multiple comparison analyses indicated that *APOE4* impaired learning of the Barnes maze test, and this impairment was worse in male compared to female *APOE4* TR mice. *APOE4* TR mice took significantly more time to complete the Barnes maze compared to *APOE3* TR mice on training days 1–3 (*p*_1_ ≤ 0.001, *p*_2_ ≤ 0.001, *p*_3_ = 0.030) in males and on training day 1 in females (*p* = 0.010). Among *APOE4* TR mice, males exhibited greater latency to goal compared to females on days 1–4 (*p*_1_ ≤ 0.001, *p*_2_ ≤ 0.001, *p*_3_ = 0.006, *p*_4_ = 0.027). Distance traveled during the test showed similar trends as latency. Recall of the target hole to assess memory of the Barnes maze arena was not impacted by *APOE* genotype, diet, or sex at 6 months old, 2 months after diet initiation (Figure 2B). Learning at 6 and 8 months old and recall at 8 months old were also unaffected by *APOE* genotype, diet, or sex (Figure S2).

To determine if mice that did not complete the maze during our pre‐diet learning assessment were driving our results, we performed repeated measures analyses with these mice removed. This did not significantly change our conclusion for the first few training days. *APOE4* TR mice had greater latency to goal compared to *APOE3* TR mice on training days 1–2 (*p*_1_ ≤ 0.001, *p*_2_ = 0.005) in males and on training day 1 in females (*p* = 0.003). Male *APOE4* TR mice had greater latency to goal versus female *APOE4* TR mice on days 1 and 2 (*p*_1_ = 0.005, *p*_2_ ≤ 0.001). To factor in mice that did not complete the maze into our model, we also compared escape distributions using pairwise log‐rank comparisons (Figure S3, Table S1). This showed that escape distributions differed in *APOE4* and *APOE3* TR mice in males on training days 1 and 2 (*p*_1_ ≤ 0.001, *p*_2_ = 0.003) and in females on training day 1 (*p*_1_ = 0.001). Escape curves were shifted to the right in *APOE4* versus *APOE3* TR mice for these comparisons, which is consistent with impaired Barnes maze performance in *APOE4* TR mice. Escape distributions also differed between male and female *APOE4* TR mice for training days 1–4 (*p*_1_ ≤ 0.001, *p*_2_ ≤ 0.001, *p*_3_ = 0.012, *p*_4_ = 0.005), which is observed as a rightwards shift in male versus female *APOE4* escape curves.

### 3.3 Apolipoprotein E4 ( *APOE4* )‐Mediated Hippocampal Enriched Proteomic Effects Depend on Sex and Diet

We next examined the proteome in hippocampal coronal tissue (enriched but not pure hippocampal tissue) to determine diet‐ and sex‐dependent effects of *APOE4* on protein expression. Across all samples, we detected 5647 proteins. Analysis of these proteins showed that enriched pathways included those involved in metabolism, synaptic communication, axonal growth, and protein synthesis (Figure S4A). Tubulin alpha‐1B chain (TUBA1B), β‐actin (ACTB), myelin proteolipid protein (PLP1), myelin basic protein (MBP), dynamin GTPase (DNM1), fructose‐bisphosphate aldolase A (ALDOA), calcium/calmodulin‐dependent protein kinase type II subunit alpha (CAMK2A), and synapsin‐1 (SYN1) represented several of the top ~1% of most abundant proteins detected across samples. This is consistent with previous data demonstrating that these same proteins are among the most highly expressed proteins in mouse brain tissue [34].

**FIGURE 3:**
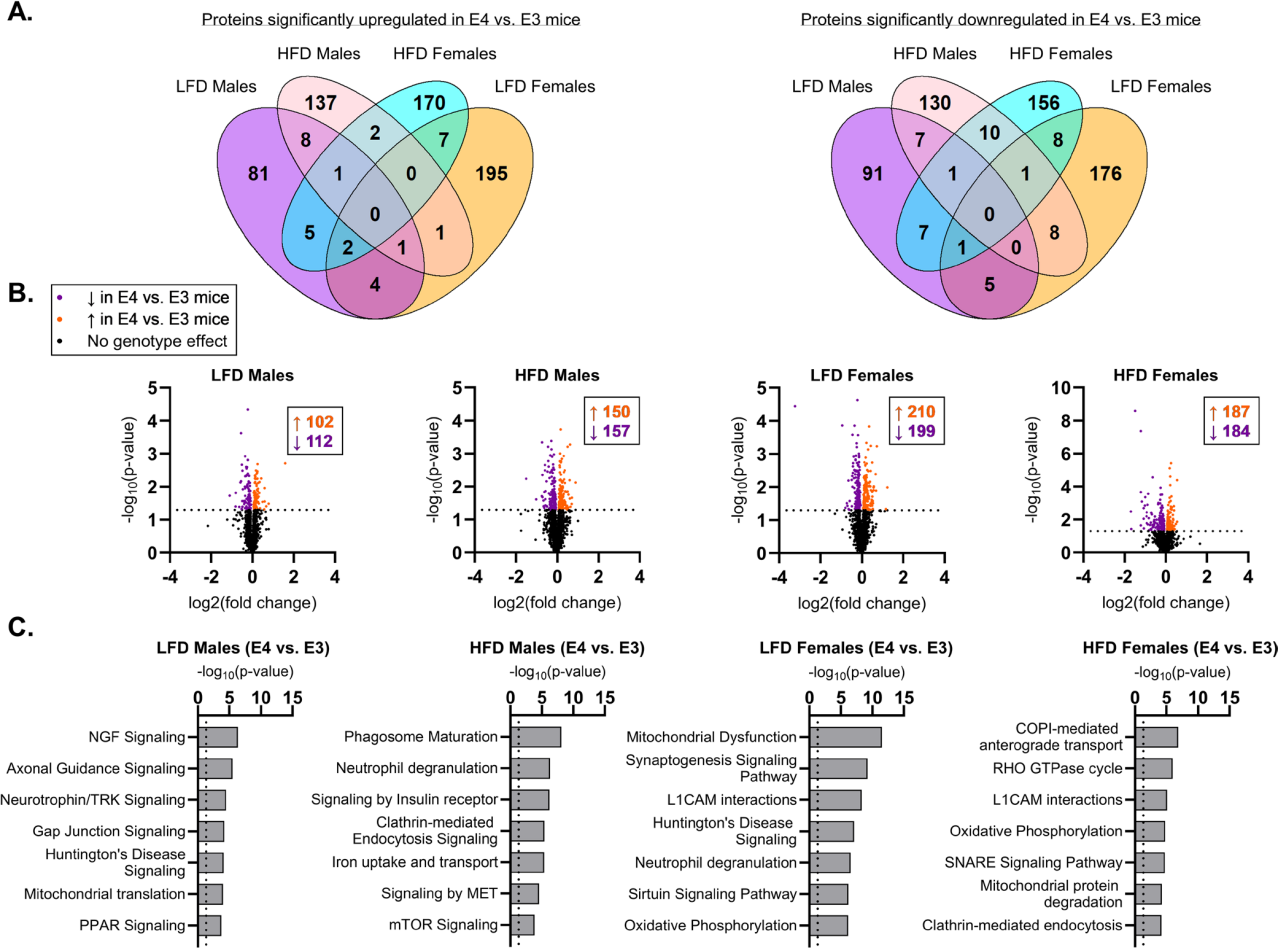
Apolipoprotein E4 (*APOE4*)‐mediated proteomic effects depend on sex and diet. Venn diagrams displaying the number of proteins significantly upregulated or downregulated (*p* ≤ 0.05) in 8‐month‐old *APOE4* versus *APOE3* targeted replacement (TR) mice and the overlap in *APOE4*‐driven effects across groups (A). Volcano plots graphing −log_10_(*p* value) versus log_2_(fold change) for difference in protein expression in 8‐month‐old *APOE4* versus *APOE3* TR mice (B). Bar graphs showing −log_10_(*p* value) for several pathways in the top 20 of most enriched pathways based on analysis of significantly altered proteins in 8‐month‐old mice (C). Vertical dotted line is drawn at significance threshold (*p* = 0.05). Sample size: *n* = 4/group. LFD, low‐fat diet; HFD, high‐fat diet; E3, apolipoprotein E3; E4, apolipoprotein E4.

After evaluating overall protein abundance and pathway enrichment, we analyzed only proteins that differed between *APOE3* and *APOE4* TR mice (Figure 3). We found that *APOE4* is associated with altered expression of 214 proteins in LFD males, 307 proteins in HFD males, 409 proteins in LFD females, and 371 proteins in HFD females (Figure 3A,B). Most proteins that were either increased or decreased by *APOE4* were uniquely altered in each sex and diet group (Figure 3A). No individual protein expression was significantly altered with a false discovery rate applied. Pathway analysis of these proteins demonstrated substantial variation among top pathways affected in each group (Figure 3C, Table S2), providing additional evidence that *APOE4* exerts sex‐ and diet‐dependent proteomic effects. Despite the heterogeneity of *APOE4*‐mediated alterations, we found that among these top pathways, *APOE4* affected processes related to mitochondrial function in several groups. This included mitochondrial translation in LFD males, mitochondrial dysfunction and oxidative phosphorylation in LFD females, and oxidative phosphorylation in HFD females.

### 3.4 Apolipoprotein E4 ( *APOE4* ) Impacts Mitochondrial Protein Expression in a Sex‐ and Diet‐Dependent Manner

**FIGURE 4:**
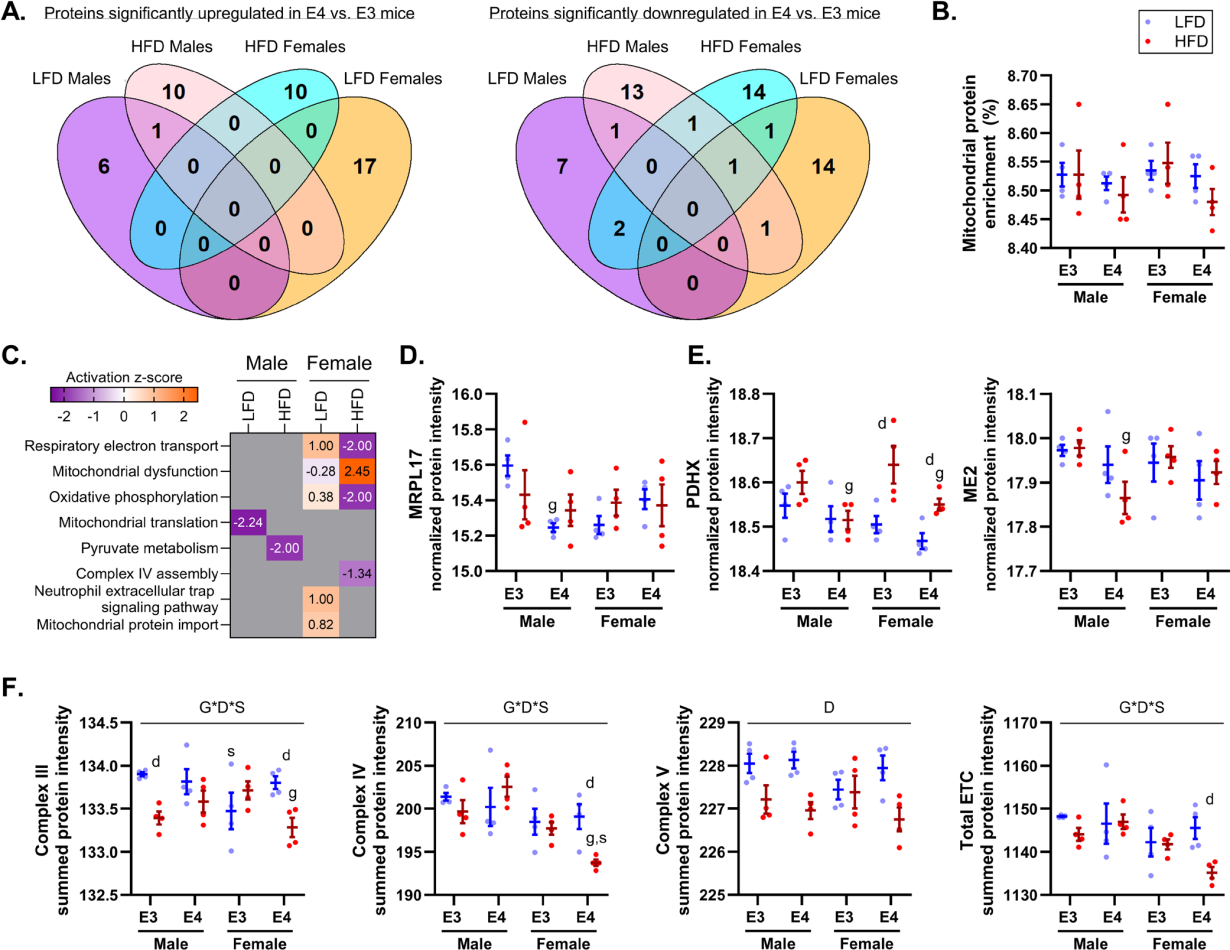
Apolipoprotein E4 (*APOE4*) impacts mitochondrial protein expression in a sex‐ and diet‐dependent manner. MitoCarta3.0 was used to identify mitochondrial proteins present in our dataset from 8‐month‐old mice. Venn diagrams displaying the number of mitochondrial proteins significantly upregulated or downregulated (*p* ≤ 0.05) in 8‐month‐old *APOE4* versus *APOE3* targeted replacement (TR) mice and the overlap in *APOE4*‐driven effects across groups (A). Scatter dot plot showing the percent enrichment of mitochondrial proteins relative to the entire proteome in 8‐month‐old mice (B). Heatmap of activation z‐scores for mitochondrial pathways significantly affected by *APOE4* (*p* ≤ 0.05) in 8‐month‐old mice. Gray boxes indicate pathways that failed to meet the enrichment *p* value cut off (C). Significant activation *z*‐scores are considered < −2 (downregulated) or > 2 (upregulated). Scatter dot plot of mitochondrial ribosomal protein L17 (MRPL17), representing one of the proteins involved in mitochondrial translation in 8‐month‐old mice (D). Pyruvate dehydrogenase complex component X (PDHX) and malic enzyme 2 (ME2), representing two proteins that are part of the pyruvate metabolism pathway in 8‐month‐old mice (E). Summed expression of all proteins associated with electron transport chain (ETC) complex III, IV, V and total ETC expression in 8‐month‐old mice (F). All protein intensities are presented as variance stabilization normalization (VSN) log_2_ values with mean ± SEM. The following symbols represent significant (*p* ≤ 0.05) comparisons: G*D*S, genotype by diet by sex interaction; D, main effect diet; g, genotype effect within diet/sex condition; d, diet effect within genotype/sex condition; s, sex effect within genotype/diet condition. Sample size: *n* = 4/group. LFD, low‐fat diet; HFD, high‐fat diet; E3, apolipoprotein E3; E4, apolipoprotein E4.

Since *APOE4* can induce mitochondrial dysfunction [14, 16] and mitochondrial dysfunction may drive downstream AD pathology [11, 12], we decided to further investigate mitochondrial protein changes (Figure 4). Like our observation at the level of the whole proteome, we found minimal overlap in proteins that were upregulated or downregulated in *APOE4* versus *APOE3* TR mice across groups (Figure 4A). While there were no differences in overall mitochondrial protein expression (Figure 4B), further analysis showed that mitochondrial pathways were significantly impacted by *APOE4* in all groups, although these effects depended on sex and diet (Figure 4C). Of the 1140 genes in MitoCarta3.0, we detected 443, and only 44 were significantly upregulated across genotype comparisons (9.93%), and 55 downregulated across comparisons (12.42%) with a total of 22.35% of mitochondrial proteins being differentially expressed.

In LFD males, mitochondrial translation was downregulated by *APOE4*, which was driven by reduced expression of proteins like mitochondrial ribosomal protein L17 (MRPL17) (*p* = 0.005) (Figure 4D), part of the 39S mitochondrial ribosomal subunit. Pyruvate metabolism was significantly lowered by *APOE4* in HFD males, as represented by decreased expression of pyruvate dehydrogenase complex component X (PDHX) (*p* = 0.041) and malic enzyme 2 (ME2) (*p* = 0.029) in HFD *APOE4* versus *APOE3* TR males (Figure 4E). Interestingly, PDHX was also reduced by *APOE4* in HFD females (*p* = 0.037) and was increased by HFD in *APOE3* (*p* = 0.002) and *APOE4* (*p* = 0.039) TR females. PDHX protein forms the E3 binding protein subunit of the pyruvate dehydrogenase complex, which is responsible for mitochondrial oxidation of pyruvate. While several mitochondrial pathways met criteria for significant alteration between *APOE4* and *APOE3* TR mice in LFD females, including oxidative phosphorylation and mitochondrial protein import, there were no pathways with significant activation *z*‐scores (< −2 or > 2) to predict the direction of pathway activity. This is likely due to changes in protein expression that were inconsistent with a specific direction of pathway activity. In contrast, we found that several pathways exhibited significant activation *z*‐scores in HFD females. Respiratory electron transport and oxidative phosphorylation were downregulated, while mitochondrial dysfunction was upregulated in HFD *APOE4* versus *APOE3* TR females.

Since further investigation showed that changes in electron transport chain (ETC) protein subunits were largely responsible for the multiple mitochondrial pathways affected by *APOE4* in HFD females, we compared the expression of ETC complexes across groups by summing the expression of all subunits for each ETC complex per sample (Figure 4F). Only two of the four subunits of complex II were identified in the entire dataset and only one of these proteins was detected in every sample, so we did not analyze complex II expression. There was a significant interaction between genotype, diet, and sex on complex III (*p* = 0.006), IV (*p* = 0.026) and total ETC (*p* = 0.046) expression. HFD *APOE4* TR females displayed reduced expression of complex III (*p* = 0.020) and complex IV (*p* = 0.038), and a trend toward lower total ETC (*p* = 0.069) relative to HFD *APOE3* TR females. HFD was also linked to lower expression of several ETC complexes. Complex V was decreased in HFD versus LFD mice (main effect diet, *p* ≤ 0.001). In *APOE3* TR males, complex III levels were also decreased by HFD (*p* = 0.007). In *APOE4* TR females, HFD was also tied to lower complex III (*p* = 0.006), IV (*p* = 0.007), and total ETC (*p* = 0.006) expression.

### 3.5 Apolipoprotein E4 ( *APOE4* ) is Linked to Lower Basal Mitochondrial Respiration in Females

**FIGURE 5:**
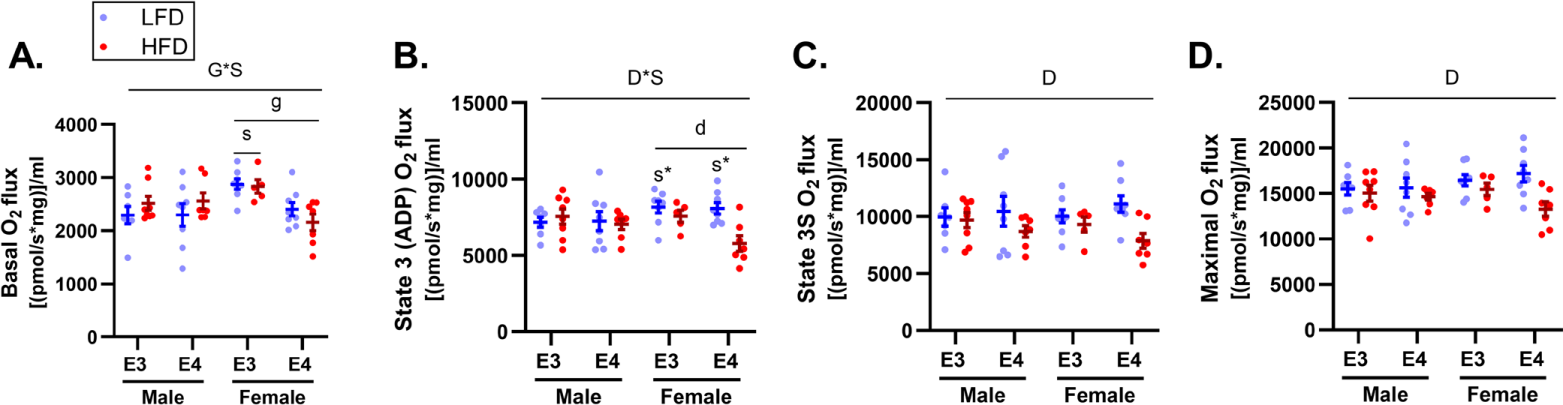
Apolipoprotein E4 (*APOE4*) is linked to lower basal mitochondrial respiration in females. Pyruvate‐supported oxygen consumption was measured in mitochondria isolated from the whole left‐brain hemisphere in 8‐month‐old mice under basal (A), state 3 (ADP) (B), and maximal (FCCP) (C) respiratory conditions. Values are presented as mean ± SEM. The following symbols represent significant (*p* ≤ 0.05) comparisons: G*S, genotype by sex interaction; D*S, diet by sex interaction; (D) main effect diet; g, genotype effect within sex condition; d, diet effect within sex condition; s, sex effect within genotype condition; s*, sex effect within genotype/diet condition. Sample size: *n* = 5–8/group. LFD, low‐fat diet; HFD, high‐fat diet; E3, apolipoprotein E3; E4, apolipoprotein E4.

To establish if *APOE4*‐mediated differences in levels of mitochondrial proteins involved in pyruvate oxidation and ETC activity translated to functional effects, we assessed pyruvate‐supported respiration in isolated brain mitochondria. Under basal respiratory conditions, there was a significant interaction between *APOE* genotype and sex on mitochondrial oxygen consumption (*p* = 0.008) (Figure 5A). Post hoc analyses showed lower basal respiration in *APOE4* versus *APOE3* TR mice in females only (*p* ≥ 0.001). Basal respiration was also lower in male versus female *APOE3* TR mice (*p* = 0.005). Under state 3 (ADP‐stimulated) conditions, we observed a significant interaction between diet and sex on mitochondrial respiration (*p* = 0.022) (Figure 5B). This was driven by lower state 3 mitochondrial respiration in HFD versus LFD mice in females only (*p* = 0.002) and in male versus female LFD mice (*p* = 0.048). Analysis of state 3S (succinate) and maximal respiration revealed reduced respiration in HFD versus LFD mice (main effect diet, *p*_state3S_ = 0.012, *p*_maximal_ = 0.008) (Figure 5C,D).

## 4 Discussion

While preclinical models indicate that Western dietary habits may worsen metabolic and cognitive impairment associated with *APOE4* [19, 20, 21], less is known about the modulatory role of sex and corresponding changes in AD pathology and proteins that may influence this relationship. Here, we showed that *APOE4* drives reductions in pathways that support mitochondrial energy production in mouse hippocampal enriched coronal tissue in a sex‐ and diet‐dependent manner. This occurred with reduced basal mitochondrial respiration in *APOE4* versus *APOE3* TR female mice. These changes occurred without differences in AD biomarkers between *APOE4* and *APOE3* TR mice, implicating a potential role for sex‐ and diet‐dependent mitochondrial alterations in *APOE4*‐linked AD pathogenesis. It is important to note that the results presented here are preliminary and require further follow‐up with higher powered studies.

Serum GFAP reflects astrocyte activation associated with inflammation and serum NfL reflects neuronal damage [35, 36]. While both biomarkers increase with age, they are also elevated in several neurodegenerative diseases, including during the preclinical stages of AD [37]. In our study, sex and diet independently affected serum GFAP, while *APOE4* did not impact biomarker levels. Although unaffected by sex at 6 months old, GFAP levels increased in males and decreased in females from 6 to 8 months, explaining significantly higher serum GFAP values in males versus females at 8 months old. Diet showed the reverse effect, where serum GFAP was elevated in HFD versus LFD mice at 6 months old yet unaffected by diet at 8 months old. This was associated with a rise in GFAP levels in LFD mice and a concomitant drop in GFAP levels in HFD mice from 6 to 8 months old. A similar diet effect was observed for NfL, where body mass‐adjusted serum NfL levels were greater in HFD versus LFD mice at 6 months old only. This suggests that HFD may acutely activate astrocytic inflammation and induce neuronal damage, regardless of genotype, and these effects likely dampen over time. Overall, our findings indicate that male sex and HFD may drive neurodegeneration and inflammation regardless of *APOE* genotype.

Effects of diet, sex, and genotype were observed across AD‐like pathological outcomes. Of note, *APOE4* males and females had the greatest effects across comparisons. These differences included altered pTau396 levels, with *APOE4* males showing a trend toward higher levels than *APOE3* males. Similar results have been previously reported in a pre‐clinical mouse model where *APOE4* increases tau phosphorylation [38]. Notably, a sex effect within genotype emerged, as *APOE4* females on a HFD exhibited lower pTau396 levels than their male counterparts. When examining amyloid levels, we found that *APOE4* mice showed higher Aβ_40_ accumulation in the brain, with changes further influenced by both diet and sex, and *APOE4* females on a HFD showing the greatest increase. However, results for Aβ_42_ differed, with more specific changes observed in *APOE4* HFD males, who exhibited increased levels. The Aβ_42_/_40_ ratio was also increased in *APOE4* HFD males, indicating greater accumulation of Aβ_42_, an effect that was alleviated by a LFD. In contrast, *APOE4* females showed a different pattern; while they demonstrated increased Aβ40, they had decreased Aβ_42_ and a lower Aβ_42_/_40_ ratio. Although these results are not entirely unsurprising, they demonstrate that amyloid is influenced not only by *APOE* genotype but also by interactions with sex and diet, which is consistent with other studies [39, 40]. Finally, levels of APOE in the brains of these mice were decreased by the *APOE4* genotype and slightly increased by a high‐fat diet. When correlating Aβ_42_ with APOE levels, we observed a positive correlation exclusively in *APOE4* mice. This was an interesting finding and indicates that while APOE is lower in the brains of *APOE4* animals, levels of the protein increase with Aβ42 and not in *APOE3* mice.

Although *APOE4* did not impact AD biomarkers, we found that learning was impaired in *APOE4* versus *APOE3* TR mice prior to diet start at 3 months old. This is consistent with existing evidence that *APOE4* mice exhibit cognitive deficits at this age [41]. Interestingly, this effect was stronger in males, which is surprising because males are thought to be less impacted by *APOE4* than females in terms of AD risk [23]. Regardless, this underscores the importance of including both sexes when evaluating *APOE4*‐mediated effects on cognition. Our observation that *APOE4* and sex did not affect memory or learning at 6 or 8 months old may be explained by several reasons. At the first timepoint, mice were exposed to the Barnes maze arena for the first time. This contrasts with later timepoints, where mice were already accustomed to the Barnes maze procedure, which could have masked subtle group differences in performance. This explanation is congruent with findings that *APOE4* is linked to poorer cognitive function at later ages [20, 22]. Future studies could initiate the first Barnes maze test at an older age or consider the use of other cognitive tests at later time points. Alternatively, the lack of group differences in Barnes maze performance at later timepoints may reflect an ability to compensate for *APOE4*‐mediated effects on cognitive function at these ages. To note, a major limitation is the small group sizes examined. These small group sizes limit power and detection of potentially biologically relevant changes in memory and learning.

The hippocampus is one of the earliest brain regions affected in AD. Our discovery that most proteins in this region that are either increased or decreased by *APOE4* are uniquely affected in each group suggests that sex and diet are important determinants of *APOE4*‐mediated pathogenic effects in this area. While we did not purify the hippocampus region with dissection, we did enrich for hippocampus. It is important to note that proteomic findings also have cortical and thalamus regions in the hippocampal enriched samples. Findings that mitochondrial pathways were significantly reduced by *APOE4* in a sex‐ and diet‐dependent manner support existing evidence that *APOE4* induces mitochondrial dysfunction [14, 16], while revealing the potential mediating roles of sex and diet in this relationship. Interestingly, we found that PDHX expression, a component of PDH, was decreased in male and female *APOE4* versus *APOE3* TR mice on a HFD only. Given its role in converting pyruvate into acetyl‐CoA for downstream oxidation in the citric acid cycle, PDH plays a critical role in mitochondrial energy production. PDH activity is reduced in brain tissue from individuals with AD [42] and is affected by *APOE4* in preclinical models [43, 44]. Our data indicate that diet may serve as a key player in this relationship, but further studies are required with higher numbers of animals to increase power and determine true effect sizes. It is important to note that no individual protein values were significant with FDR during proteomics analysis and thus these findings are discovery based and require further validation.

In addition to PDH, the activity and expression of mitochondrial ETC complexes are also affected by *APOE4* carrier status and AD [45, 46, 47, 48], possibly contributing to energy deficits observed in AD. Our assessment of mitochondrial ETC complexes corroborated these findings by showing that *APOE4* is associated with lower complex III and IV expression in HFD females. This is in line with work in neuronal cell models, demonstrating that proteolytic cleavage fragments of *APOE4* interact with and reduce the activity of these same ETC components [49]. Our study indicates that sex and diet may further impact this association. Our results also suggest that HFD impairs mitochondrial ETC expression in all mice, supported by our finding that HFD is associated with reduced complex V levels regardless of *APOE* genotype and sex‐ and genotype‐dependent reductions in other ETC complexes.

Respirometry analyses in isolated mitochondria revealed that *APOE4* was only linked to both molecular and functional mitochondrial changes in HFD females. While average mitochondrial respiration was lowest in *APOE4* TR females across all tested conditions, this only translated to a significant effect of *APOE* genotype during basal respiration, an effect that was observed regardless of diet. Similar studies with older mice would help determine if sex‐ and diet‐dependent changes in mitochondrial protein expression mediated by *APOE4* translate to pronounced deficits in mitochondrial function over time, particularly under conditions that promote ETC activity (state 3). Repeated studies in younger mice may also inform the temporal relationship between cognitive and mitochondrial deficits. In addition to *APOE* genotype, our finding that HFD was associated with lower state 3 mitochondrial respiration in females and state 3S and maximal respiration in both sexes implicates diet as an important effector of bioenergetic function that works independently of *APOE* genotype. These functional differences in respiratory function could be driven by the observed reduction in ETC expression in HFD versus LFD mice. Our data warrant further investigation into the early role of dietary habits on brain metabolic function prior to the onset of cognitive impairment.

There are several strengths of our study. We used multiple robust methods to comprehensively assess molecular and functional effects of *APOE4*. For detection of serum biomarkers, we used the Simoa platform, which is considered an ultrasensitive state‐of‐the‐art immunoassay and is commonly employed in AD research. To test cognitive function, we used the Barnes maze procedure, a tool that is widely implemented in rodent AD studies to evaluate hippocampal‐dependent memory and learning. While the lack of stressful stimuli could affect motivation to complete the Barnes maze, we accounted for this in our analysis. Additionally, we chose this test over the Morris water maze because the latter procedure is more stressful and *APOE* genotype is associated with differences in anxiety behavior in HFD‐fed mice [21, 50]. The value of our study is also enhanced by the direct assessment of molecular outcomes in brain tissue. We used proteomics to comprehensively compare protein expression between groups and interpreted our findings in the context of functional mitochondrial changes, assessed with high‐resolution respirometry. This allowed us to interrogate multiple aspects of mitochondrial respiratory function. While we acknowledge that proteomic analyses in hippocampal coronal sections and respirometry analyses in mitochondria from the whole left‐brain hemisphere limit our interpretation, the ability to collect enough hippocampal tissue for both proteomics and respirometry is challenging.

While there are strengths in our study, there are also weaknesses. The first weakness is limited power and group sizes when comparing data across genotype, sex, and diet. Despite this, our goal is to provide our findings in the context of multiple complementary outcomes to provide a resource for hypothesis generation and testing in the context of *APOE* and AD risk. The data provided here are useful for power calculations and mechanistic study designs for future larger studies. Therefore, it is important to note that proteomic data requires further replication in future larger study cohorts with stringent false discovery rate cutoffs. We focused on *APOE3* and *APOE4* and did not consider the effects of *APOE2*. Future studies should also consider *APOE2*, especially in the context of sex and diet, given its implications in cardiovascular health. It is also important to note that expressing human APOE in mice could impact biological function and findings. The results of this study should be regarded in the greater context of human‐based studies leveraging clinical cohorts and human cell lines for increased rigor.

In conclusion, we provide novel evidence that *APOE4* mediates early sex‐ and/or diet‐dependent differences in cognitive performance, protein expression, and mitochondrial function in mice. While more work is needed to understand if these findings play an etiologic role in AD, the immediate impact of our work underscores the need to consider both sex and diet when studying the effects of *APOE4*. Notably, diet is a modifiable factor that may induce early‐life mitochondrial alterations that could contribute to AD pathogenesis.

## Author Contributions

Formal analysis: C.N.J., C.R.L., X.C., V.C., F.B., P.J.K., and E.F. Acquisition of funding: P.C.G., J.K.M., J.P.T., and H.M.W. Writing – original draft preparation: C.R.L. and C.N.J. Writing – review and editing: all authors.

## Conflicts of Interest

The authors declare no conflicts of interest.

## Supporting information


**Table S1:** Comparison of pre‐diet Barnes maze escape distributions among apolipoprotein E (*APOE*) genotype and sex groups. Log‐rank Mantal–Cox tests were performed to compare Kaplan–Meier curves of escape percentage as a function of escape latency for each training day. Pairwise log‐rank comparisons were used to compare escape distributions among different genotype/sex combinations if the curves were significantly different (log‐rank *p* value, *p* ≤ 0.05).
**Table S2:** Top 20 proteomic pathways that differ between apolipoprotein E4 (*APOE4*) and *APOE3* targeted‐replacement mice among sex and diet groups ranked by enrichment *p* values.

**Figure S1:** Brain Dissection. Brain matrix showing razor blade placement (red lines) and cuts to enrich for hippocampal coronal sections used in proteomics analyses.
**Figure S2:** Learning assessment at 6 and 8 months old and recall test at 8 months old. Spatial learning was evaluated with the Barnes maze procedure at 6 months old (A) and recall of the target hole to assess memory was tested at 8 months old, 2 months after diet initiation (B). Spatial learning was assessed again at 8 months old (C). Latency to goal and distance traveled are presented as mean ± SEM for each training day. Sample size: *n* = 3–4/group. LFD, low‐fat diet; HFD, high‐fat diet; E3, apolipoprotein E3; E4, apolipoprotein E4.
**Figure S3:** Barnes maze escape distributions for pre‐diet assessment in 3‐month‐old mice. Kaplan–Meier curves of escape percentage as a function of escape latency are shown for each training day in males (A) and females (B). Sample size: *n* = 3–4/group. E3, apolipoprotein E3; E4, apolipoprotein E4.
**Figure S4:** Protein abundance and pathway enrichment in hippocampal coronal tissue sections in 8‐month‐old mice. Bar graph displaying the‐log_10_(*p* value) for the top 40 most significantly enriched pathways based on analysis of all detected proteins across all groups (A). Horizonal dotted line is drawn at significance threshold (*p* = 0.05). Scatter dot plots of select proteins from the top ~1% of most abundant proteins detected across samples based on average protein expression (B). Values are presented as mean ± SEM. The following symbols represent significant (*p* ≤ 0.05) comparisons: g, genotype effect within diet/sex condition; d, diet effect within genotype/sex condition. Sample size: *n* = 4/group. LFD, low‐fat diet; HFD, high‐fat diet; E3, apolipoprotein E3; E4, apolipoprotein E4. Gene symbols for proteins: TUBA1B, tubulin alpha‐1B chain; ACTB, β‐actin; PLP1, myelin proteolipid protein; MBP, myelin basic protein; DNM1, dynamin GTPase; ALDOA, fructose‐bisphosphate aldolase A; CAMK2A, calcium/calmodulin‐dependent protein kinase type II subunit alpha; SYN1, synapsin‐1.

## Data Availability

Research data are available upon request from the corresponding author.
