# Supplementary material for: APOE4 Drives Sex‐ and Diet‐Dependent Effects on AD‐Like Pathology, Cognition, and Mitochondrial Function
Source: FASEB Bioadv. 2026 May 6;8(5):e70113. doi: 10.1096/fba.2026-00121 (PMC13146351; doi:10.1096/fba.2026-00121)
Supplement: Supplementary file 1 — Table S1: Comparison of pre‐diet Barnes maze escape distributions among apolipoprotein E (APOE) genotype and sex groups. Log‐rank Mantal–Cox tests were performed to compare Kaplan–Meier curves of escape percentage as a function of escape latency for each training day. Pairwise log‐rank comparisons were used to compare escape distributions among different genotype/sex combinations if the curves were significantly different (log‐rank p value, p ≤ 0.05). Table S2: Top 20 proteomic pathways that differ between apolipoprotein E4 (APOE4) and APOE3 targeted‐replacement mice among sex and diet groups ranked by enrichment p values. [file FBA2-8-e70113-s002.docx]

**Supplemental table 1. Comparison of pre-diet Barnes maze escape distributions among apolipoprotein E (*APOE*) genotype and sex groups.** Log-rank Mantal-Cox tests were performed to compare Kaplan-Meier curves of escape percentage as a function of escape latency for each training day. Pairwise log-rank comparisons were used to compare escape distributions among different genotype/sex combinations if the curves were significantly different (log-rank p-value p≤0.05).

|  | **Median latency** | | | | | **Log-rank pairwise comparison p-values** | | | |
| --- | --- | --- | --- | --- | --- | --- | --- | --- | --- |
| **Training day** | **E3 males** | **E4 males** | **E3 females** | **E4 females** | **Log-rank p-value** | **Genotype effect within males** | **Genotype effect within females** | **Sex effect within E3s** | **Sex effect within E4s** |
| 1 | 76.72 | 147.36 | 56.10 | 93.98 | <0.001* | <0.001* | 0.001* | 0.217 | <0.001* |
| 2 | 34.94 | 111.82 | 33.69 | 24.30 | <0.001* | 0.003* | 0.392 | 0.427 | <0.001* |
| 3 | 33.44 | 67.57 | 20.27 | 35.90 | 0.005* | 0.083 | 0.435 | 0.102 | 0.012* |
| 4 | 23.94 | 39.32 | 21.17 | 12.46 | 0.015* | 0.672 | 0.659 | 0.187 | 0.005* |
| 5 | 19.48 | 26.54 | 16.22 | 16.02 | 0.457 | N/A | N/A | N/A | N/A |

**Supplemental table 2. Top 20 proteomic pathways that differ between apolipoprotein E4 (*APOE4*) and *APOE3* targeted-replacement mice among sex and diet groups ranked by enrichment p-values.**

| **Ingenuity Canonical Pathway** | **-log(p-value)** | **z-score** |
| --- | --- | --- |
| **LFD males E4 vs E3** | | |
| NGF Signaling | 6.35 | 0.333 |
| GNRH Signaling | 5.55 | -0.333 |
| Axonal Guidance Signaling | 5.51 | N/A |
| Neurotrophin/TRK Signaling | 4.49 | 0.447 |
| Signaling by NTRK1 (TRKA) | 4.40 | 1.633 |
| UVC-Induced MAPK Signaling | 4.33 | -0.447 |
| Gap Junction Signaling | 4.18 | 0.905 |
| CD27 Signaling in Lymphocytes | 4.13 | -0.447 |
| CNTF Signaling | 4.13 | 1.342 |
| HGF Signaling | 4.13 | -0.378 |
| Huntington's Disease Signaling | 4.06 | 1.134 |
| ERBB Signaling | 4.06 | 0 |
| Mitochondrial translation | 3.96 | -1.633 |
| 4-1BB Signaling in T Lymphocytes | 3.87 | 0 |
| Colanic Acid Building Blocks Biosynthesis | 3.81 | N/A |
| PTEN Signaling | 3.74 | -0.378 |
| PPAR Signaling | 3.70 | -0.816 |
| Cardiac Hypertrophy Signaling | 3.67 | -0.707 |
| ERK5 Signaling | 3.56 | -0.447 |
| Protein methylation | 3.54 | N/A |
| **HFD males E4 vs E3** | | |
| Phagosome Maturation | 8.08 | N/A |
| Neutrophil degranulation | 6.32 | 3.13 |
| Signaling by Insulin receptor | 6.21 | 1.134 |
| Clathrin-mediated Endocytosis Signaling | 5.36 | N/A |
| Iron uptake and transport | 5.34 | 1.89 |
| EIF2 Signaling | 4.79 | -1.00 |
| COPII-mediated vesicle transport | 4.77 | 0.378 |
| Cargo recognition for clathrin-mediated endocytosis | 4.63 | -1.414 |
| Signaling by MET | 4.54 | -1.134 |
| Role of MAPK Signaling in Promoting the Pathogenesis of Influenza | 4.37 | 1.89 |
| Regulation of eIF4 and p70S6K Signaling | 4.28 | -1.342 |
| CLEAR Signaling Pathway | 4.03 | -1.155 |
| Pyrimidine Ribonucleotides Interconversion | 4.00 | N/A |
| Clathrin-mediated endocytosis | 3.99 | -0.707 |
| Processing of Capped Intron-Containing Pre-mRNA | 3.99 | -0.577 |
| Nephrin family interactions | 3.95 | -2.00 |
| GM-CSF Signaling | 3.89 | -1.342 |
| Pyrimidine Ribonucleotides De Novo Biosynthesis | 3.85 | N/A |
| mTOR Signaling | 3.82 | -0.816 |
| Reelin Signaling in Neurons | 3.79 | -0.378 |
| **LFD females E4 vs E3** | | |
| Mitochondrial Dysfunction | 11.5 | -0.200 |
| Synaptogenesis Signaling Pathway | 9.26 | -0.426 |
| Nonsense-Mediated Decay (NMD) | 8.59 | 3.207 |
| Coronavirus Replication Pathway | 8.34 | N/A |
| L1CAM interactions | 8.26 | 2.138 |
| MHC class II antigen presentation | 8.08 | 2.138 |
| Signaling by ROBO receptors | 7.41 | 1.633 |
| Selenoamino acid metabolism | 7.34 | 3.742 |
| Response of EIF2AK4 (GCN2) to amino acid deficiency | 7.32 | 3.464 |
| Eukaryotic Translation Initiation | 7.20 | 2.673 |
| Huntington's Disease Signaling | 7.10 | 0 |
| Neutrophil degranulation | 6.55 | 0.408 |
| Eukaryotic Translation Elongation | 6.50 | 3.464 |
| Eukaryotic Translation Termination | 6.50 | 3.464 |
| Sirtuin Signaling Pathway | 6.19 | -2.333 |
| Clathrin-mediated Endocytosis Signaling | 6.17 | N/A |
| Oxidative Phosphorylation | 6.16 | 0 |
| SRP-dependent cotranslational protein targeting to membrane | 5.79 | 3.464 |
| Ribosomal Quality Control Signaling Pathway | 5.57 | 4 |
| COPI-mediated anterograde transport | 5.50 | 1.667 |
| **HFD females E4 vs E3** | | |
| COPI-mediated anterograde transport | 6.85 | 2.714 |
| RHO GTPase cycle | 5.98 | 3.273 |
| L1CAM interactions | 5.12 | 1.897 |
| Cytoprotection by HMOX1 | 4.89 | -1.633 |
| Oxidative Phosphorylation | 4.78 | -2.646 |
| SNARE Signaling Pathway | 4.71 | 0.333 |
| Semaphorin interactions | 4.60 | 1.890 |
| Netrin-1 signaling | 4.57 | 1.633 |
| Keratinization | 4.38 | -2.530 |
| Complex IV assembly | 4.35 | -1.633 |
| Mitochondrial protein degradation | 4.25 | -2.121 |
| Clathrin-mediated endocytosis | 4.17 | 1.667 |
| trans-Golgi Network Vesicle Budding | 4.15 | 0.378 |
| Mitochondrial Dysfunction | 4.09 | 1.941 |
| Netrin Signaling | 3.84 | 0.000 |
| Fatty Acid Biosynthesis Initiation II | 3.74 | N/A |
| Tight Junction Signaling | 3.72 | N/A |
| NCAM signaling for neurite out-growth | 3.68 | 1.633 |
| RHOGDI Signaling | 3.66 | -1.134 |
| Nephrin family interactions | 3.62 | 1.000 |
