# Supplementary figures and images for: APOE4 Drives Sex‐ and Diet‐Dependent Effects on AD‐Like Pathology, Cognition, and Mitochondrial Function
Source: FASEB Bioadv. 2026 May 6;8(5):e70113. doi: 10.1096/fba.2026-00121 (PMC13146351; doi:10.1096/fba.2026-00121)

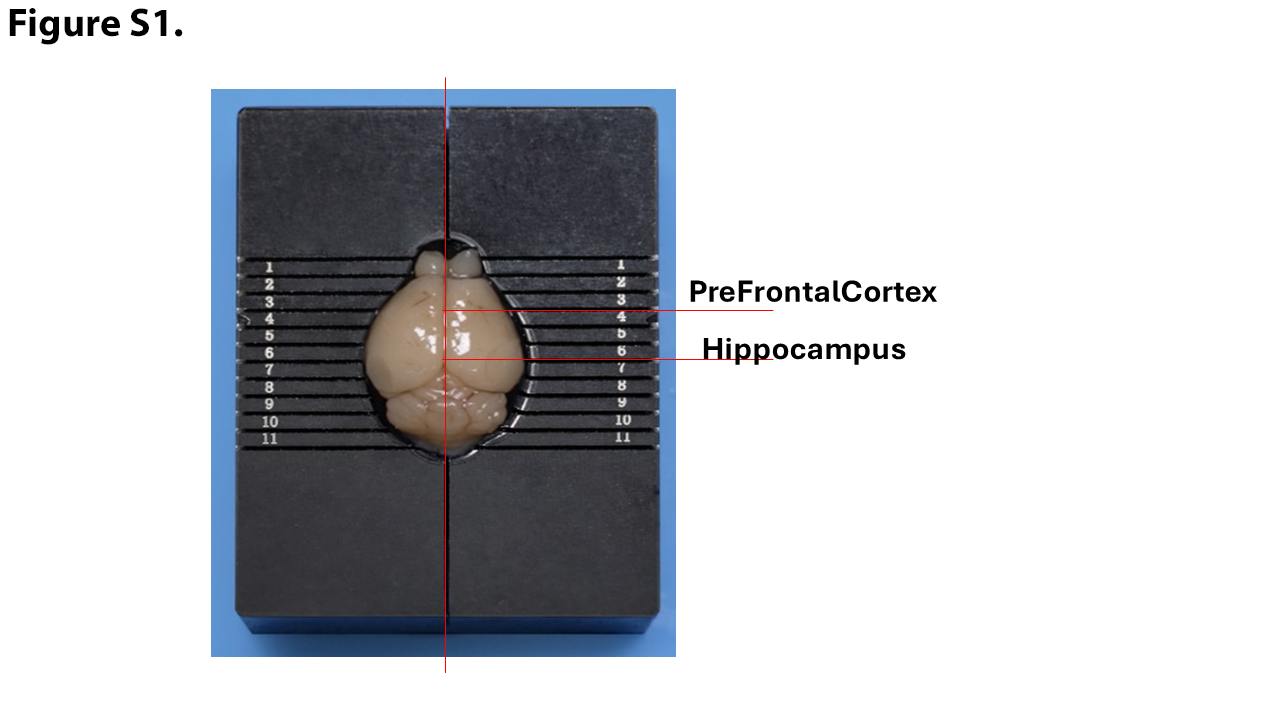

Supplement: Supplementary file 2 — Figure S1: Brain Dissection. Brain matrix showing razor blade placement (red lines) and cuts to enrich for hippocampal coronal sections used in proteomics analyses. Figure S2: Learning assessment at 6 and 8 months old and recall test at 8 months old. Spatial learning was evaluated with the Barnes maze procedure at 6 months old (A) and recall of the target hole to assess memory was tested at 8 months old, 2 months after diet initiation (B). Spatial learning was assessed again at 8 months old (C). Latency to goal and distance traveled are presented as mean ± SEM for each training day. Sample size: n = 3–4/group. LFD, low‐fat diet; HFD, high‐fat diet; E3, apolipoprotein E3; E4, apolipoprotein E4. Figure S3: Barnes maze escape distributions for pre‐diet assessment in 3‐month‐old mice. Kaplan–Meier curves of escape percentage as a function of escape latency are shown for each training day in males (A) and females (B). Sample size: n = 3–4/group. E3, apolipoprotein E3; E4, apolipoprotein E4. Figure S4: Protein abundance and pathway enrichment in hippocampal coronal tissue sections in 8‐month‐old mice. Bar graph displaying the‐log10(p value) for the top 40 most significantly enriched pathways based on analysis of all detected proteins across all groups (A). Horizonal dotted line is drawn at significance threshold (p = 0.05). Scatter dot plots of select proteins from the top ~1% of most abundant proteins detected across samples based on average protein expression (B). Values are presented as mean ± SEM. The following symbols represent significant (p ≤ 0.05) comparisons: g, genotype effect within diet/sex condition; d, diet effect within genotype/sex condition. Sample size: n = 4/group. LFD, low‐fat diet; HFD, high‐fat diet; E3, apolipoprotein E3; E4, apolipoprotein E4. Gene symbols for proteins: TUBA1B, tubulin alpha‐1B chain; ACTB, β‐actin; PLP1, myelin proteolipid protein; MBP, myelin basic protein; DNM1, dynamin GTPase; ALDOA, fructose‐bisphosphate aldolase A; CAMK2A, ca [file FBA2-8-e70113-s001.zip › 2026-00121-T-sup-0003--S.tif]

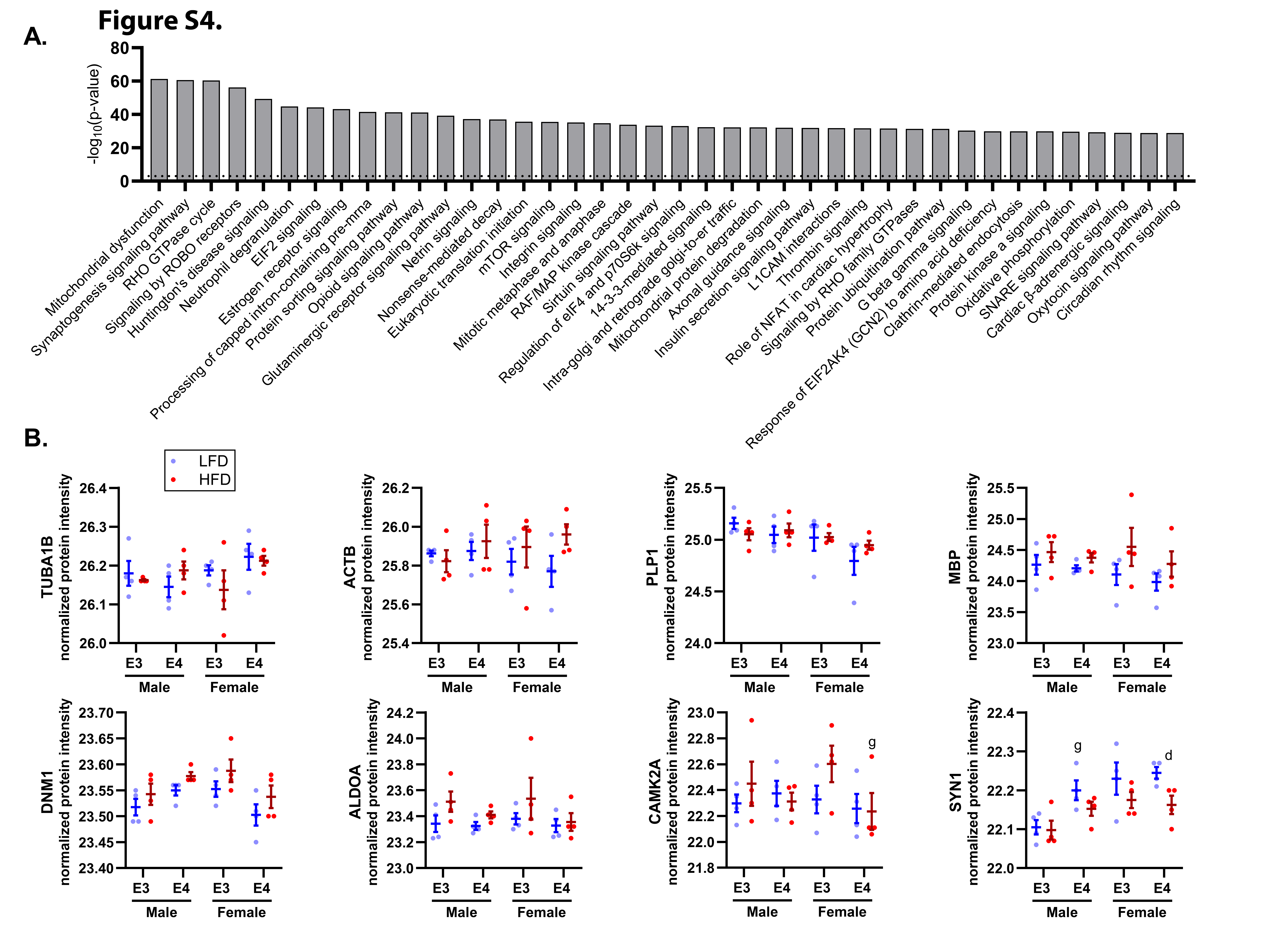

Supplement: Supplementary file 2 — Figure S1: Brain Dissection. Brain matrix showing razor blade placement (red lines) and cuts to enrich for hippocampal coronal sections used in proteomics analyses. Figure S2: Learning assessment at 6 and 8 months old and recall test at 8 months old. Spatial learning was evaluated with the Barnes maze procedure at 6 months old (A) and recall of the target hole to assess memory was tested at 8 months old, 2 months after diet initiation (B). Spatial learning was assessed again at 8 months old (C). Latency to goal and distance traveled are presented as mean ± SEM for each training day. Sample size: n = 3–4/group. LFD, low‐fat diet; HFD, high‐fat diet; E3, apolipoprotein E3; E4, apolipoprotein E4. Figure S3: Barnes maze escape distributions for pre‐diet assessment in 3‐month‐old mice. Kaplan–Meier curves of escape percentage as a function of escape latency are shown for each training day in males (A) and females (B). Sample size: n = 3–4/group. E3, apolipoprotein E3; E4, apolipoprotein E4. Figure S4: Protein abundance and pathway enrichment in hippocampal coronal tissue sections in 8‐month‐old mice. Bar graph displaying the‐log10(p value) for the top 40 most significantly enriched pathways based on analysis of all detected proteins across all groups (A). Horizonal dotted line is drawn at significance threshold (p = 0.05). Scatter dot plots of select proteins from the top ~1% of most abundant proteins detected across samples based on average protein expression (B). Values are presented as mean ± SEM. The following symbols represent significant (p ≤ 0.05) comparisons: g, genotype effect within diet/sex condition; d, diet effect within genotype/sex condition. Sample size: n = 4/group. LFD, low‐fat diet; HFD, high‐fat diet; E3, apolipoprotein E3; E4, apolipoprotein E4. Gene symbols for proteins: TUBA1B, tubulin alpha‐1B chain; ACTB, β‐actin; PLP1, myelin proteolipid protein; MBP, myelin basic protein; DNM1, dynamin GTPase; ALDOA, fructose‐bisphosphate aldolase A; CAMK2A, ca [file FBA2-8-e70113-s001.zip › 2026-00121-T-sup-0005--S.tif]

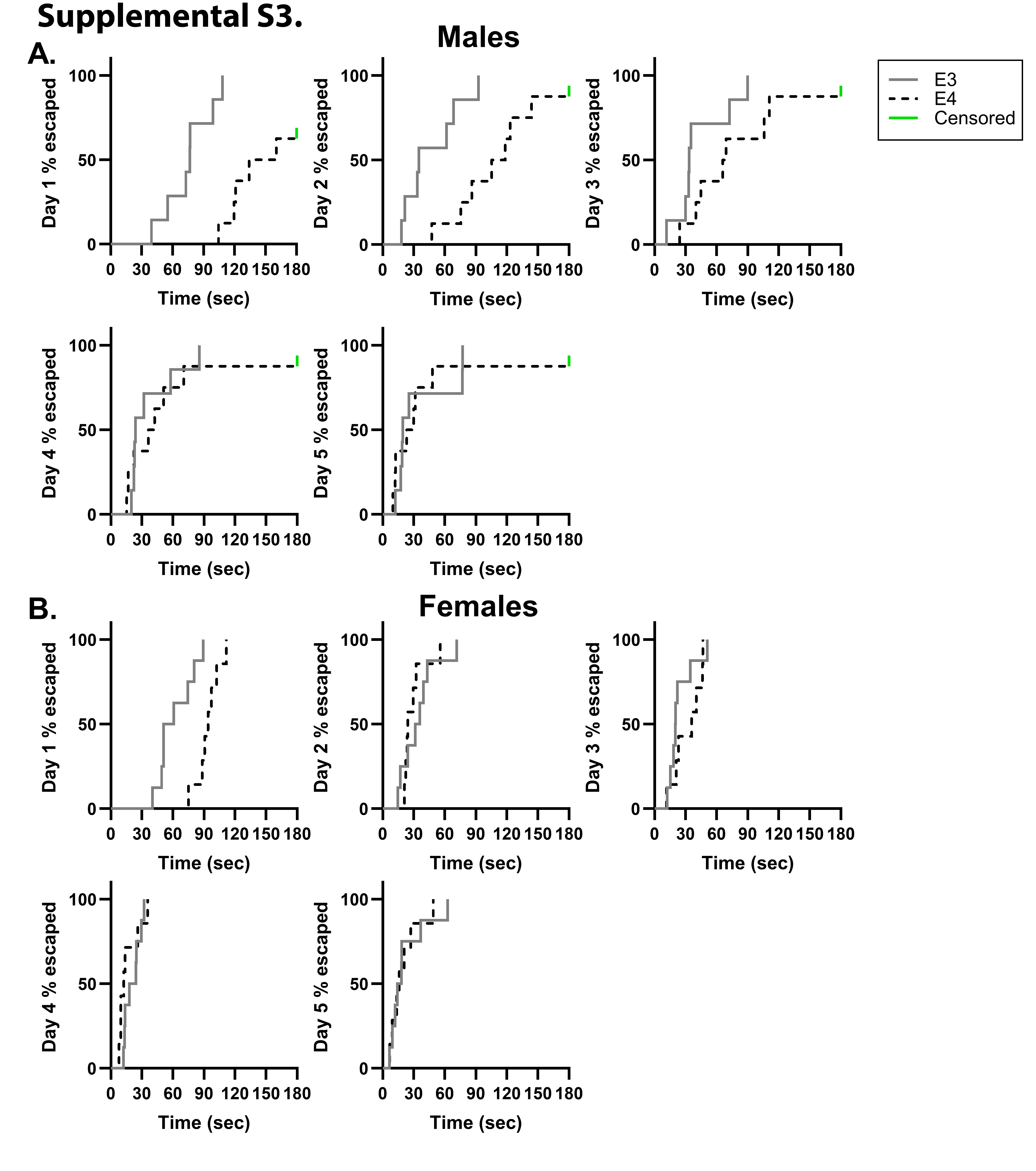

Supplement: Supplementary file 2 — Figure S1: Brain Dissection. Brain matrix showing razor blade placement (red lines) and cuts to enrich for hippocampal coronal sections used in proteomics analyses. Figure S2: Learning assessment at 6 and 8 months old and recall test at 8 months old. Spatial learning was evaluated with the Barnes maze procedure at 6 months old (A) and recall of the target hole to assess memory was tested at 8 months old, 2 months after diet initiation (B). Spatial learning was assessed again at 8 months old (C). Latency to goal and distance traveled are presented as mean ± SEM for each training day. Sample size: n = 3–4/group. LFD, low‐fat diet; HFD, high‐fat diet; E3, apolipoprotein E3; E4, apolipoprotein E4. Figure S3: Barnes maze escape distributions for pre‐diet assessment in 3‐month‐old mice. Kaplan–Meier curves of escape percentage as a function of escape latency are shown for each training day in males (A) and females (B). Sample size: n = 3–4/group. E3, apolipoprotein E3; E4, apolipoprotein E4. Figure S4: Protein abundance and pathway enrichment in hippocampal coronal tissue sections in 8‐month‐old mice. Bar graph displaying the‐log10(p value) for the top 40 most significantly enriched pathways based on analysis of all detected proteins across all groups (A). Horizonal dotted line is drawn at significance threshold (p = 0.05). Scatter dot plots of select proteins from the top ~1% of most abundant proteins detected across samples based on average protein expression (B). Values are presented as mean ± SEM. The following symbols represent significant (p ≤ 0.05) comparisons: g, genotype effect within diet/sex condition; d, diet effect within genotype/sex condition. Sample size: n = 4/group. LFD, low‐fat diet; HFD, high‐fat diet; E3, apolipoprotein E3; E4, apolipoprotein E4. Gene symbols for proteins: TUBA1B, tubulin alpha‐1B chain; ACTB, β‐actin; PLP1, myelin proteolipid protein; MBP, myelin basic protein; DNM1, dynamin GTPase; ALDOA, fructose‐bisphosphate aldolase A; CAMK2A, ca [file FBA2-8-e70113-s001.zip › 2026-00121-T-sup-0006--S.tif]

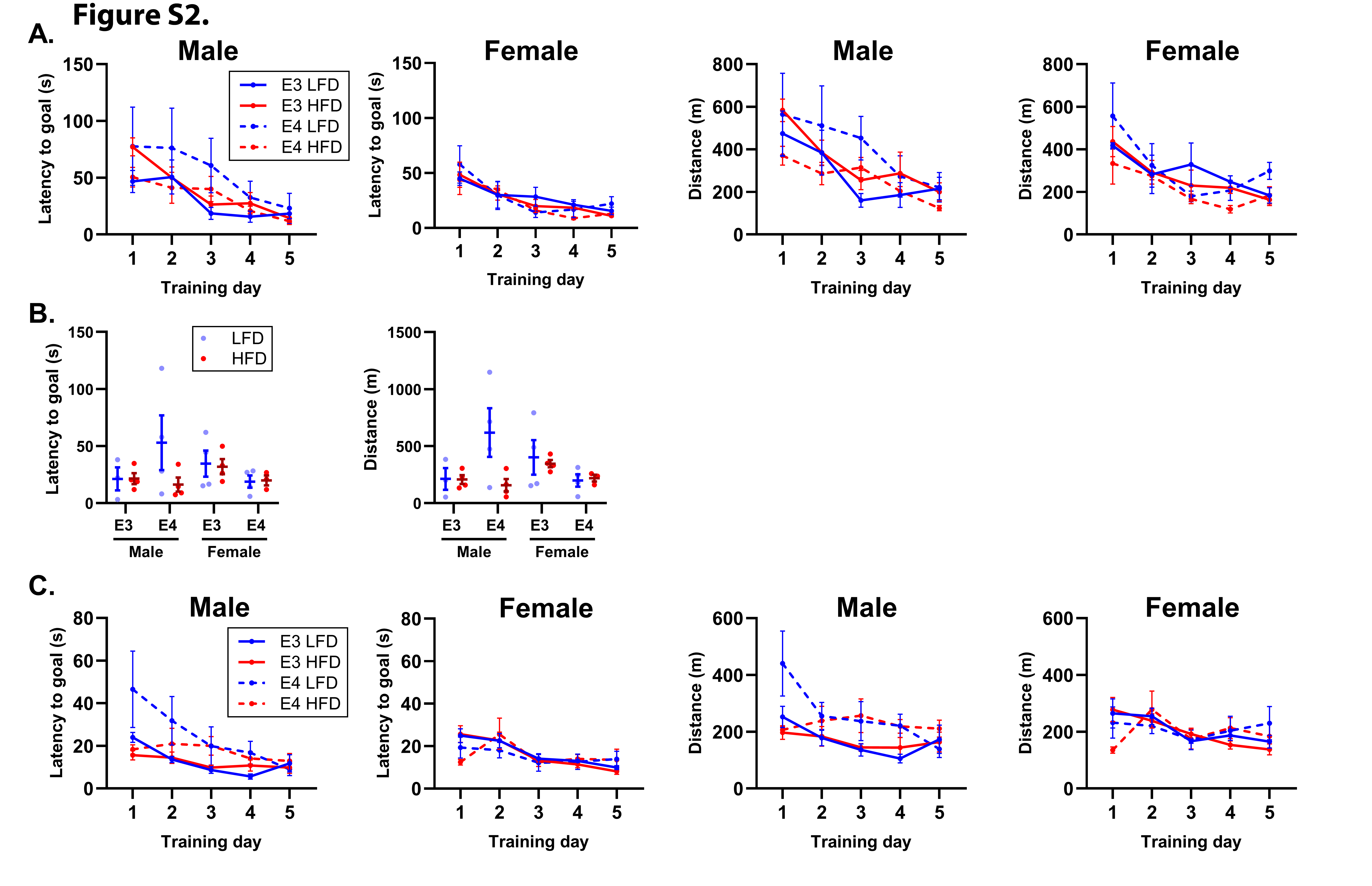

Supplement: Supplementary file 2 — Figure S1: Brain Dissection. Brain matrix showing razor blade placement (red lines) and cuts to enrich for hippocampal coronal sections used in proteomics analyses. Figure S2: Learning assessment at 6 and 8 months old and recall test at 8 months old. Spatial learning was evaluated with the Barnes maze procedure at 6 months old (A) and recall of the target hole to assess memory was tested at 8 months old, 2 months after diet initiation (B). Spatial learning was assessed again at 8 months old (C). Latency to goal and distance traveled are presented as mean ± SEM for each training day. Sample size: n = 3–4/group. LFD, low‐fat diet; HFD, high‐fat diet; E3, apolipoprotein E3; E4, apolipoprotein E4. Figure S3: Barnes maze escape distributions for pre‐diet assessment in 3‐month‐old mice. Kaplan–Meier curves of escape percentage as a function of escape latency are shown for each training day in males (A) and females (B). Sample size: n = 3–4/group. E3, apolipoprotein E3; E4, apolipoprotein E4. Figure S4: Protein abundance and pathway enrichment in hippocampal coronal tissue sections in 8‐month‐old mice. Bar graph displaying the‐log10(p value) for the top 40 most significantly enriched pathways based on analysis of all detected proteins across all groups (A). Horizonal dotted line is drawn at significance threshold (p = 0.05). Scatter dot plots of select proteins from the top ~1% of most abundant proteins detected across samples based on average protein expression (B). Values are presented as mean ± SEM. The following symbols represent significant (p ≤ 0.05) comparisons: g, genotype effect within diet/sex condition; d, diet effect within genotype/sex condition. Sample size: n = 4/group. LFD, low‐fat diet; HFD, high‐fat diet; E3, apolipoprotein E3; E4, apolipoprotein E4. Gene symbols for proteins: TUBA1B, tubulin alpha‐1B chain; ACTB, β‐actin; PLP1, myelin proteolipid protein; MBP, myelin basic protein; DNM1, dynamin GTPase; ALDOA, fructose‐bisphosphate aldolase A; CAMK2A, ca [file FBA2-8-e70113-s001.zip › 2026-00121-T-sup-0007--S.tif]
